# Mineral Balance and Metabolic Syndrome in Adolescents: Focus on Calcium and Phosphorus Intake

**DOI:** 10.3390/healthcare9111525

**Published:** 2021-11-09

**Authors:** Yoonjin Park, Jungjin Han

**Affiliations:** 1 Department of Nursing, Joongbu University, Geumsan-gun 32716, Korea; pyj2272@naver.com; 2 Department of Nursing, Semyung University, Jecheon-si 27136, Korea

**Keywords:** adolescents, metabolic syndrome, calcium, phosphorus, minerals, nutrients

## Abstract

The incidence of metabolic syndrome, a chronic disease, tends to increase in adolescence, but has not been a high priority in delivery of health services. This study was to investigate the relationship between metabolic syndrome prevalence and mineral balance such as calcium and phosphorus intake among Korean adolescents. This study is a cross-sectional descriptive study using data from the 7th Korean national health and nutrition examination survey (KNHANES) VII-3 (2018) and the 8th KNHANES VII-1 (2019). A total of 895 adolescents aged 12 to 18 who filled in mineral intake questionnaires were analyzed using SPSS. According to their responses, only 2.9% of the subjects had a calcium: phosphorus intake ratio of 1:1, which is the recommended ratio. Daily phosphorus intake was significantly correlated with systolic blood pressure (r = 0.448, *p* < 0.001), waist circumference (r = 0.115, *p* = 0.001), HDL cholesterol (r = −0.113, *p* = 0.002), and daily calcium intake (r = 0.697, *p* = 0.001). And, as the serum creatinine increased by 1, the risk of metabolic syndrome increased 16.5 times (OR: 16.452, 95% CI: 1.701–159.136, *p* < 0.05). Excessive phosphorus intake and high creatinine levels may increase the risk of metabolic syndrome in adolescents. Therefore, education is necessary to encourage adolescents to follow a balanced diet that contains essential minerals. In addition, it is suggested to expand the metabolic syndrome prevention education, which has been largely targeted towards adults.

## 1. Introduction

Metabolic syndrome is a cluster of multiple factors including abdominal obesity, hypertension, abnormal glucose metabolism, dyslipidemia, and low HDL cholesterol. It reportedly increases the risk of cardiovascular disease, diabetes, and cancer [1]. According to the Korea National Health and Nutrition Survey, the prevalence of metabolic syndrome in the last 12 years was 25.5% among those in their 40s, 17.1% among those in their 30s, and 7.1% among those ages 19 to 29, and that prevalence has been increasing overall [2]. Approximately 3.3% of children and adolescents worldwide are estimated to have metabolic syndrome [3], suggesting the need for active prevention and management of metabolic syndrome starting with adolescence. 

Despite the need for the prevention and management of metabolic syndrome, the Health Behavior and Chronic Disease Statistics released by the Korea Disease Control and Prevention Agency show that only 30% of adolescents have a balanced diet, and 16.5% consume fast food, while 35.8% drink soft drinks at least three times per week [4]. Convenient or instant foods, which are preferred by adolescents, are high in calories, fat, and sodium, while lacking in minerals essential for growth such as calcium, magnesium, and zinc [5]. They must be consumed with caution, as studies report that instant foods such as cup noodles and canned beverages can increase the serum creatinine level in children [6].

Minerals are essential for maintaining homeostasis although they do not provide energy. A daily intake of 100 mg of macrominerals and 20 mg of trace minerals is recommended. Calcium and phosphorus are major macrominerals. Ninety-nine percent of calcium exists within the bones and teeth, and 85% of phosphorus binds with calcium to constitute bones and teeth; thus, the balance between these two minerals is important [7]. Calcium and phosphorus play an important role in cell metabolism, osteogenesis, and protein synthesis [8]. Calcium is involved in the regulation of nerve impulse transmission, muscle contraction, and blood pressure, and phosphorus is involved in maintaining homeostasis and acid–base balance [9].

The guidebook Dietary Reference Intakes for Koreans recommends daily calcium and phosphorus intakes of 800–1000 mg and 1200 mg for adolescents aged 12–18 years, respectively, with a recommended ratio of 1:1 between the two minerals [10]. However, according to the KNHANES, from 2013 to 2017, the median daily intake of calcium was 489.51 mg/day for adolescents aged 12–18 years, lower than the daily recommended amount, and adolescents in the said age range accounted for the highest proportion (male and female: 91.0% and 90.5%) of the total population not meeting the daily requirement [11]. Only 10% of adolescents took calcium supplements, meaning adolescents get most of their calcium intake from food. The mean daily phosphorus intake was 1261.3 mg/day and 981.1 mg/day for male and female adolescents aged 12–14 years, respectively, and 1306.9 mg/day and 902.7 mg/day for male and female adolescents aged 15–18 years, respectively. Although the phosphorus intake among adolescents was closer to the recommended intake compared to calcium intake, the ratio between calcium and phosphorus intake was not adequate [10]. An unbalanced diet including soft drinks, which are frequently consumed by adolescents, decreases the consumption of healthy foods such as milk, thereby decreasing calcium intakes while excessively increasing phosphorus intakes [12].

Excessive phosphorus intake has a negative effect on bone metabolism by reducing calcium absorption, thereby inducing osteoporosis, and can lead to hyperphosphatemia and hyperparathyroidism [13]. High serum phosphorus levels are a risk factor for vascular calcification [14] and, if present with other risk factors, can increase the risk of and mortality rate from cardiovascular disease [15,16]. Calcium intake below the requirement can reduce bone density and mass in growing children, induce osteoporosis, and increase the risk of non-communicable chronic diseases such as hypertension, dyslipidemia, cardiovascular disease, diabetes, and cancer [17,18,19] For this reason, calcium intake may be closely associated with metabolic syndrome [20,21]. Since metabolic syndrome during adolescence can affect the risk of cardiovascular disease in adulthood, it requires early detection and management [22]. However, due to the relatively low prevalence of metabolic syndrome among adolescents compared to other population groups, adolescents receive low priority in health and medical care services for metabolic syndrome. Furthermore, the diagnostic criteria for metabolic syndrome from the International Diabetes Federation (IDF) and the National Cholesterol Education Program Adult Treatment Panel III (NCEP-ATP III) are interchangeably used to diagnose metabolic syndrome. Despite the reports that inadequate nutrition during adolescence negatively affects chronic diseases such as metabolic syndrome [6], and that metabolic syndrome is an important predictor of renal disease [23], research is still lacking on metabolic syndrome among adolescents.

Thus, in this study we used the data from the 7th KNHANES VII-3 (2018) and the 8th KNHANES VII-1 (2019) to analyze the relation between calcium and phosphorus intakes, creatinine and blood urea nitrogen (BUN), and the prevalence risk of metabolic syndrome in Korean adolescents.

## 2. Materials and Methods

### 2.1. Study Design and Participants

This is a secondary analysis study conducted with the approval to use the raw data. The data from the 7th KNHANES VII-3 (2018) and the 8th KNHANES VII-1 (2019) conducted by the Korea Disease Control and Prevention Agency were downloaded and analyzed in this cross-sectional, descriptive survey study. The KNHANES has been conducted annually since 2007. The survey is conducted through health interviews and mobile examinations and samples 23 households across 192 regions by probability sampling, surveying approximately 10,000 household members over the period of one year. The KNHANES conducted in 2019 increased the number of surveyed households to 25 and used a systemic sampling method to select participants. The 7th KNHANES VII-3 (2018) surveyed 10,453 participants. Of these, 7992 participants (76.5%) had participated in at least one of the following surveys: a health interview, health examination, and/or nutrition survey. The 8th KNHANES VII-1 (2019) surveyed 10,859 participants. Of these, 8110 participants (74.7%) had participated in at least one of the three surveys of the KNHANES. 

This study aims to analyze the correlation between calcium and phosphorus intake in adolescents and metabolic syndrome. Therefore, 893 adolescents aged 12–18 who reported daily calcium and phosphorus intake were included in the analysis.

### 2.2. Methods

#### 2.2.1. Data Collection

General characteristics including age, gender, and the components of metabolic syndrome were investigated. Body mass index (BMI), low-density lipoprotein (LDL), cholesterol, serum creatinine, and serum BUN, which are associated with metabolic syndrome, were also investigated.

#### 2.2.2. Biochemical Measurements

A blood test was performed after at least eight hours of fasting. Blood was usually collected from the median cubital vein and cephalic vein. Blood samples were stored in a fridge and analyzed within 24 h after being transported to the laboratory medicine facility. Fasting glucose, HDL cholesterol, LDL cholesterol, and triglycerides were measured via enzymatic methods using Hitachi automatic analyzer 7600 (Tokyo, Japan).

#### 2.2.3. Metabolic Syndrome

Using the definition provided by the NCEP-ATP III [1] in 2001, metabolic syndrome was defined as the presence of at least three of the following factors: (1) increased weight circumference, (2) hypertension, (3) elevated triglycerides, (4) low HDL cholesterol, and (5) impaired fasting glucose. The diagnostic criteria for abdominal obesity in Koreans proposed by the Korean Society for the Study of Obesity [24] were used. Thus, metabolic syndrome was defined as the presence of at least three of the following factors: (1) systolic blood pressure ≥130 mmHg or diastolic blood pressure ≥85 mmHg, (2) a waist circumference ≥90 cm for men and ≥85 cm for women, (3) fasting glucose ≥100 mg/dL, (4) blood triglycerides ≥150 mg/dL, and (5) blood HDL cholesterol ≤40 mg/dL for men and ≤50 mg/dL for women. For adolescent children, the diagnostic criteria for metabolic syndrome published by the IDF in 2007 were used. For adolescents aged 10–16 years, metabolic syndrome was defined as having a waist circumference ≥90% of the normal waist circumference for adults, blood triglycerides ≥150 mg/dL, HDL cholesterol <50 mg/dL, systolic blood pressure ≥130 mmHg or diastolic blood pressure ≥85 mmHg, fasting blood glucose ≥100 mg/dL, or previous diagnosis with type II diabetes. For adolescents aged ≥17 years, the diagnostic criteria for adults were used. 

#### 2.2.4. Phosphorus/Calcium Intake Ratio

The nutritional survey of the KNHANES consisted of the current status of dietary behavior, dietary supplements, nutritional knowledge, and food stability, and the contents of food intake one day before the survey (24-h recall method), and daily nutrient intake were calculated. The Dietary Reference Intakes for Koreans 2020 [10] recommends calcium intakes of 900–1000 mg/day and 800–900 mg/day for male and female adolescents, respectively, and a phosphorus intake of 1200 mg/day for both genders. In this study, correlations between calcium and phosphorus intakes and metabolic syndrome were analyzed by dividing participants into groups with varying ratios of calcium-to-phosphorus intakes (1:1, 1:2, 1:3, 1:4, and 1:5). 

#### 2.2.5. Statistical Analysis

SPSS version 22 (IBM Co., Armonk, NY, USA) was used for data analysis with a significance level of *p*-value < 0.05. Since regions (enumeration districts) are sampled by stratified multi-stage cluster sampling, which is a complex sampling method, weights were applied to each region using the SPSS Complex Samples module. The weight of the KHNANES is an expanded multiplier given that an estimate represents the entire population of South Korea and is calculated by reflecting the extraction and response rates and the population distribution. If a new variable was produced by combining several variables or a statistical model was constructed using several variables, the survey sections, domains, and items of all variables were considered in order to choose appropriate weights. Weights that encompass several survey sections, domains, and items were named “weights for correlation analysis” and presented for each year. 

Differences in the calcium/phosphorus ratio according to general characteristics were analyzed using ANOVA and a t-test. To identify predictive risk factors of metabolic syndrome, the subcategories of metabolic syndrome (waist circumference; high blood pressure; abnormal triglyceride level; abnormal HDL cholesterol; and abnormal fasting glucose level) and phosphorus and calcium intakes were analyzed by binomial regression. Pearson’s correlations were used to identify correlations among the factors.

## 3. Results

### 3.1. Demographic and Disease Characteristics

This study included 893 participants, comprising 470 males and 423 females. The mean age of the male and female participants was 14.81 and 14.96 years, respectively. The mean waist circumference was 63.55 cm for males and 69.08 cm for females, and the mean BMI was 22.07 for males and 21.08 for females. The mean systolic & diastolic blood pressure of male and female was 112.36 mmHg, 68.31 mmHg and 105.62 mmHg, 67.13 mmHg, respectively. Also, the mean of blood sugar was 92.89 mg/dL for males and 91.21 mg/dL for females. The mean serum creatinine level was 0.77 mg/dL for males and 0.62 mg/dL for females, and the mean BUN level was 13.51 mg/dL for males and 11.67 mg/dL for females. The mean calcium intake was 599.86 mg/day for males and 475.37 mg/day for females. The mean phosphorus intake was 1271.34 mg/day for males and 938.41 mg/day for females. Of the respondents, 23 males (5.9%) and 20 females (4.7%) had metabolic syndrome (Table 1). 

### 3.2. Differences in General Variables According to Ratios between Calcium and Phosphorus Intakes

Participants were divided into groups according to the calcium/phosphate intake ratio (1:1, 1:2, 1:3, 1:4, and 1:5). The mean age of the participants in each group was 15.04, 14.64, 14.98, 15.10, and 15.32 years, respectively. The mean serum creatinine level (in mg/dL) was 0.74 ± 0.135, 0.68 ± 0.150, 0.71 ± 0.145, 0.71 ± 0.155, and 0.72 ± 0.155, respectively, with significant differences between the groups (*p* < 0.05). The mean daily phosphorus intake (in mg/day) was 852.08 ± 487.406, 1087.51 ± 521.773, 1133.21 ± 498.45, 1186.24 ± 659.936, and 1144.61 ± 605.653, respectively. The group with a calcium/phosphorus ratio of 1:4 had the lowest calcium intake, which was significantly lower than the calcium intakes in the other groups (*p* < 0.05) (Table 2).

### 3.3. Correlation Analysis

The correlations between the components of metabolic syndrome—systolic and diastolic blood pressure, waist circumference, fasting glucose, triglycerides, and HDL cholesterol—and daily calcium and phosphorus intakes were analyzed. Daily phosphorus intake was significantly positively correlated with systolic blood pressure (r = 0.103, *p* < 0.004), waist circumference (r = 0.115, *p* = 0.001), daily calcium intake (r = 0.697, *p* < 0.001) and negatively correlated with HDL cholesterol (r = −0.113, *p* = 0.002). 

Daily calcium intake was significantly positively correlated with daily phosphorus intake but not with any components of metabolic syndrome (*p* > 0.05) (Table 3).

### 3.4. Predictors of Metabolic Syndrome

A regression analysis was performed using systolic blood pressure, waist circumference, HDL cholesterol, and serum creatinine, which were significantly correlated with the components of metabolic syndrome and daily phosphorus intake, as the predictor variables. As serum creatinine increased by 1, the risk of metabolic syndrome increased 16.452-fold (OR: 16.452, 95% CI: 1.701–159.136, *p* < 0.05). The relative risk of metabolic syndrome based on diastolic blood pressure, waist circumference, and HDL cholesterol was 0.983, 0.982, and 1.007, respectively; these variables were not significant predictors of metabolic syndrome (*p* > 0.05) (Table 4).

## 4. Discussion

The present descriptive survey study analyzed the 7th KNHANES VII-3 (2018) and the 8th KNHANES VII-1 (2019) to investigate the correlations between metabolic syndrome and mineral imbalance in adolescents. 

The incidence of metabolic syndrome among the adolescents included in this study was 5.9% for males and 4.7% for females. This incidence was lower than the prevalence of metabolic syndrome (10.1%) reported for adolescents aged 12–19 years in the 2001–2010 National Health and Nutrition Examination Survey in the United States [25], as well as that (6.3%) reported for Asian countries [3]. 

In this study, the participants’ calcium intake did not meet the recommended calcium intake; on the other hand, phosphorus intake met the recommended level. Also, only 2.9% of all participants had a calcium/phosphorus ratio of 1:1 (2.1% of males, 3.7% of females). Most participants (43.9%) had a calcium/phosphorus ratio of 1:2, and 8.6% of all participants had a ratio of 1:4, indicating a severe calcium/phosphorus imbalance among the participants. This result is closely associated with the dietary habits of adolescents. Soft drinks and sports drinks, commonly enjoyed by adolescents, contain up to 4.2 times and 48.5 times more phosphorus than calcium, respectively [26]. Of the 57,303 adolescents in the 15th Online Youth Health Behavior Survey (2019), 50.7% consumed soft drinks at least three times per week [27], supporting the results of this study that high phosphorus intake ratio compares to calcium.

In the analysis of correlations between calcium and phosphorus intakes and the components of metabolic syndrome, no significant correlations were found between calcium intake and the components of metabolic syndrome. Phosphorus intake was significantly positively correlated with systolic blood pressure and waist circumference and significantly negatively correlated with HDL cholesterol. Phosphorus intake was also significantly positively correlated with calcium intake (*p* < 0.05). A previous study reported elevated phosphorus levels as a risk factor for cardiovascular disease in adults. A study analyzing the Third National Health and Nutrition Examination Survey (NHANES III) (1988–1994) reported that the joint effect of elevated phosphorus levels and hypertension increases the mortality due to cardiovascular disease [15]. In a study on adults aged 40–59 years in Asia, Europe, and the United States, phosphorus intake was directly proportional to systolic and diastolic blood pressure [28]. A similar result was obtained in this study in which phosphorus intake was positively correlated with systolic blood pressure, confirming that high phosphorus intake increases the risk of metabolic syndrome. A study analyzing data from 88,094 Americans aged ≥17 years reported that the prevalence of obesity increased with serum phosphorus levels [29]. A study analyzing computed tomography (CT) results of 2509 healthy Korean adults found that the risk of coronary calcification increased with serum phosphorus levels [30], a finding that was consistent with the results of this study. Although the present study examined adolescents aged 12–18 years, it is meaningful that similar results were observed between this study and others that examined adults or elderly subjects, since health problems during adolescence such as pediatric obesity have been traditionally considered to be risk factors of metabolic syndrome [3]. This is because these results indicate that similar risk factors are at play between adolescents and adults in cases of metabolic syndrome and that it is closely associated with long-term health problems.

A significant difference in serum creatinine levels was found according to the calcium/phosphorus ratio. Creatinine is a metabolite of creatinine in muscles or a waste product of proteins. It is an important indicator of renal function alongside BUN. The normal range of creatinine is 0.5–1.4 mg/dL. Since creatinine is produced in muscles, it is found in higher levels among individuals with high muscle mass such as younger individuals than among the elderly and in higher levels among males than among females [31]. The serum creatinine levels in this study were within the normal range for both male and female participants (0.77 mg/dL and 0.62 mg/dL, respectively). However, a binomial logistic regression analysis revealed a 16-fold increase in the risk of metabolic syndrome for every 1 mg/dL increase in the serum creatinine level (*p* < 0.05). Although the exact mechanism by which serum creatinine levels increase the risk of metabolic syndrome is unclear, a previous study found that elevated creatinine levels increased six-fold the mortality of patients who had undergone heart surgery or had had heart disease [31]. A study following up on 7690 middle-aged men with no unusual conditions for an average of 14 years also reported that elevated serum creatinine levels are a risk factor of cardiovascular disease [32]. A Framingham Heart study analyzing serum creatinine levels of subjects without heart or renal disease found a correlation between hypertension, which is a component of metabolic syndrome, and BMI [33], and another study suggested serum creatinine as a predictor for the risk of metabolic syndrome [34]. In addition, the results of this study also showed that blood creatinine level is a factor that increases the risk of metabolic syndrome, which can predict the relationship between increased blood creatinine level and cardiovascular disease. The results of this study demonstrate that a continued imbalance between calcium and phosphorus intakes can potentially affect serum creatinine levels even in adolescents and act as a risk factor for metabolic syndrome, suggesting the need to monitor the intakes of these minerals in adolescents. 

However, since the participants of this study were adolescents, who are a relatively healthy population group with a lower incidence of metabolic syndrome, it was difficult to accurately examine the effect of calcium intake, necessitating a future follow-up study. This study has limitations in that time-series analysis could not be done due to the cross-sectional descriptive design of the study, and the relationships between the risk of metabolic syndrome and factors such as the serum phosphorus or calcium level could not be inferred thoroughly. Also, there is a limit to calculating long-term intake. In addition, since the reference values of adolescents are very different among the various countries, interpretation of the results should be made with caution. 

## 5. Conclusions

This is the first study to explore the relationship between calcium, phosphorus intake and the prevalence of metabolic syndrome among Korean adolescents using Korean na-tional survey data. This study confirmed that the calcium and phosphorus intake imbalance of adolescents was serious, and in particular, excessive phosphorus intake was associated with the risk of metabolic syndrome. The results of this study suggest the necessity of education to emphasize balanced diet and to reduce the consumption of soft drinks and instant foods. In addition to that, existing metabolic syndrome prevention and management programs targeted at adults should expand their target population to adolescents to preemptively reduce the prevalence of metabolic syndrome starting with adolescence. This study is meaningful in that it determines the correlations between metabolic syndrome and calcium or phosphorus intakes in adolescents, who have not been the priority of metabolic syndrome prevention efforts. Continued research on the relationship between mineral intake and metabolic syndrome in adolescents is recommended as well as development of health promotion programs aimed at preventing metabolic syndrome among adolescents.

## Figures and Tables

**Table 1:** General Characteristics of Participants (N = 893).

Category	N (%) or M ± SD	
	Male (N = 470)	Female (N = 423)
Age	14.81 ± 2.05	14.96 ± 2.05
Waist circumference (cm)	63.55 ± 15.32	69.08 ± 8.61
Height (cm)	169.05 ± 8.87	160.02 ± 6.36
Weight (kg)	63.55 ± 15.32	54.11 ± 11.28
BMI	22.07 ± 4.28	21.08 ± 3.94
Blood pressure		
Systolic (mmHg)	112.36 ± 10.32	105.62 ± 8.57
Diastolic (mmHg)	68.31 ± 9.33	67.13 ± 8.52
Fasting glucose (mg/dL)	92.89 ± 7.38	91.21 ± 7.67
Serum total cholesterol (mg/dL)	159.13 ± 26.79	166.52 ± 27.20
HDL cholesterol (mg/dL)	49.15 ± 9.37	53.24 ± 10.35
Triglyceride (mg/dL)	91.23 ± 55.78	89.17 ± 46.26
Serum creatinine (mg/dL)	0.77 ± 0.15	0.62 ± 0.09
Serum BUN (mg/dL)	13.51 ± 3.20	11.67 ± 2.83
Phosphorus intake (mg/day)	1271.34 ± 594.53	938.41 ± 418.75
Calcium intake (mg/day)	599.86 ± 350.80	475.37 ± 298.55
Metabolic syndrome prevalence (%)	23(5.9)	20(4.7)

BMI: body mass index; HDL: high density lipoprotein; BUN: blood urea nitrogen.

**Table 2:** Comparison of General Characteristics among the Calcium/Phosphorus intake ratio (N = 893).

Category	Calcium/Phosphorus Intake Ratio					F/χ^2^	*p*
	≤1:1	≤1:2	≤1:3	≤1:4	>1:5		
Age	15.04 ± 2.01	14.64 ± 2.03	14.98 ± 2.05	15.10 ± 2.06	15.32 ± 2.06	2.758	0.027
Gender							
Male	10	197	155	65	43	1.264	0.868
Female	16	196	121	56	34		
Waist circumference (cm)							
Male	81.90 ± 12.47	74.13 ± 12.15	75.51 ± 10.21	76.97 ± 11.96	77.13 ± 12.68	1.748	0.139
Female	68.02 ± 6.59	69.53 ± 8.24	68.82 ± 9.47	68.58 ± 8.49	68.68 ± 8.94	0.250	0.910
BMI	22.75 ± 5.36	21.43 ± 4.02	21.60 ± 4.07	21.64 ± 4.11	22.03 ± 4.71	0.785	0.535
Blood pressure							
Systolic (mmHg)	110.61 ± 11.75	109.32 ± 10.46	108.55 ± 9.40	109.98 ± 10.13	109.09 ± 10.22	0.543	0.704
Diastolic (mmHg)	70.13 ± 9.14	67.91 ± 8.58	67.48 ± 8.73	67.15 ± 9.00	68.10 ± 11.42	0.632	0.640
Fasting glucose (mg/dL)	90.52 ± 6.40	92.56 ± 7.97	92.11 ± 73.32	90.73 ± 7.03	92.57 ± 7.27	1.484	0.205
Serum total cholesterol (mg/dL)	163.81 ± 29.13	163.56 ± 28.75	160.41 ± 25.19	160.15 ± 25.04	168.60 ± 28.40	1.531	0.191
HDL cholesterol (mg/dL)							
Male	50.00 ± 8.36	50.44 ± 9.28	48.10 ± 8.92	49.02 ± 10.22	47.23 ± 9.78	1.664	0.157
Female	54.93 ± 13.70	52.83 ± 10.83	53.07 ± 9.94	52.06 ± 8.08	57.43 ± 10.08	1.469	0.211
Triglyceride (mg/dL)	78.57 ± 31.79	89.27 ± 47.10	89.82 ± 52.57	90.99 ± 55.12	99.45 ± 66.61	0.842	0.499
Serum creatinine (mg/dL)	0.74 ± 0.13	0.68 ± 0.15	0.71 ± 0.14	0.71 ± 0.15	0.72 ± 0.15	2.472	0.043 *
Serum BUN (mg/dL)	11.48 ± 3.66	12.41 ± 3.05	12.96 ± 3.32	12.66 ± 3.20	13.16 ± 2.85	2.221	0.065
Phosphorus intake (mg/day)	852.08 ± 487.40	1087.51 ± 521.77	1133.21 ± 498.45	1186.24 ± 659.93	1144.61 ± 605.65	2.442	0.045 *
Calcium intake (mg/day)	928.18 ± 541.79	694.56 ± 349.15	458.82 ± 203.31	343.25 ± 185.80	233.99 ± 123.70	83.887	<0.001 *
Metabolic syndrome prevalence	3	18	15	5	2	4.869	0.301

* *p* < 0.05; post-hoc Scheffe test was done. No significance was shown between groups.

**Table 3:** Correlation for BP, WC, Glucose, TG, HDL and daily phosphorus intake (N = 893).

	BPsys	BPdia	WC	Glucose	HDL	TG	Phosphorus Intake	Calcium Intake
**BPsys**	1							
**BPdia**	0.448(<0.001) *	1						
**WC**	0.482(<0.001) *	0.209(<0.001) *	1					
**glucose**	0.129(<0.001) *	0.035(0.340)	0.153(<0.001) *	1				
**HDL**	−0.169(<0.001) *	−0.107(0.003) *	−0.352(<0.001) *	−0.063(0.082)	1			
**TG**	0.203(<0.001) *	0.139(<0.001) *	0.325(<0.001) *	0.167(<0.001) *	−0.361(<0.001) *	1		
**Phosphorus intake**	0.103(0.004) *	−0.005(0.891)	0.115(0.001) *	0.020(0.575)	−0.113(0.002) *	−0.020(0.579)	1	
**Calcium intake**	0.058(0.100)	−0.004(0.907)	0.049(0.163)	0.029(0.425)	−0.063(.079)	−0.041(0.251)	0.697(<0.001) *	1

BPsys: Systolic Blood Pressure, BPdia: Diastolic Blood Pressure, WC: Waist circumference, HDL: High Density Lipoprotein, TG: Triglyceride. * *p* < 0.05.

**Table 4:** Risk Factors for Metabolic Syndrome (N = 893).

Category	B	S.E.	Wald	df	*p*	Exp (B)	95% C.I.	
							Lower	Upper
Systolic BP (mmHg)	−0.007	0.020	0.115	1	0.735	0.993	0.955	1.033
Waist circumference (cm)	−0.018	0.021	0.735	1	0.391	0.982	0.943	1.023
HDL cholesterol (mg/dL)	0.007	0.018	0.129	1	0.719	1.007	0.971	1.043
Serum creatinine (mg/dL)	2.800	1.158	5.850	1	0.016 *	16.452	1.701	159.136
Constant	−3.161	2.381	1.762	1	0.184	0.042		

HDL: High Density Lipoprotein, TG: Triglyceride. * *p* < 0.05.

## Data Availability

Not applicable.
